# And, not or: Quality, quantity in scientific publishing

**DOI:** 10.1371/journal.pone.0178074

**Published:** 2017-06-01

**Authors:** Matthew J. Michalska-Smith, Stefano Allesina

**Affiliations:** 1 Department of Ecology & Evolution, University of Chicago, Chicago IL, United States of America 60637; 2 Computation Institute, University of Chicago, Chicago IL, United States of America 60637; 3 Northwestern Institute on Complex Systems, Northwestern University, Evanston IL, United States of America 60208; International Nutrition Inc, UNITED STATES

## Abstract

Scientists often perceive a trade-off between quantity and quality in scientific publishing: finite amounts of time and effort can be spent to produce few high-quality papers or subdivided to produce many papers of lower quality. Despite this perception, previous studies have indicated the opposite relationship, in which productivity (publishing more papers) is associated with increased paper quality (usually measured by citation accumulation). We examine this question in a novel way, comparing members of the National Academy of Sciences with themselves across years, and using a much larger dataset than previously analyzed. We find that a member’s most highly cited paper in a given year has more citations in more productive years than in in less productive years. Their lowest cited paper each year, on the other hand, has fewer citations in more productive years. To disentangle the effect of the underlying distributions of citations and productivities, we repeat the analysis for hypothetical publication records generated by scrambling each author’s citation counts among their publications. Surprisingly, these artificial histories re-create the above trends almost exactly. Put another way, the observed positive relationship between quantity and quality can be interpreted as a consequence of randomly drawing citation counts for each publication: more productive years yield higher-cited papers because they have more chances to draw a large value. This suggests that citation counts, and the rewards that have come to be associated with them, may be more stochastic than previously appreciated.

## Introduction

A common perception among scientists is that there exists a trade-off between quality and quantity in scientific publishing: one can either spend a long time working on a high-quality paper or write many papers of lower quality, each taking a fraction of the time [1–5]. This idea was recently encapsulated by Sarewitz [6], stating that “Scientists must publish less, or good research will be swamped by the ever-increasing volume of poor work.” Historically, however, the opposite relationship, *i*.*e*. a concordant increase of quality with quantity, has been observed in a number of disciplines [4, 7–10], with some authors even purporting that a high level of output is a *necessary* condition for making high-quality contributions [11, 12].

Within the scientific community, one can find pressures on both fronts: to publish both more *and* better [4]. These pressures are, in part, a result of the varied and complicated incentive structures at play. Differences in the reward system for scientists have been observed to be a key driver of publication practices and policies [3, 11, 13, 14], even to the point of subconsciously igniting reward pathways in the brain [15]. Yet, these systems can often have unintended consequences. For example, systems based on number of publications can disincentivize the pursuit of risky or long-term research which might necessitate a period of low research output. Similarly, systems which reward high citation rates can disincentivize the expansion into new fields of research, especially underpopulated ones, where there is less opportunity for one’s publications to be cited by others [16].

This conflict is apparent in job applications as well. Applications which require a listing of all publications put an emphasis on the number of articles published—“the length of one’s vita” [1]. Alternatively, some organizations are moving toward just requesting the few most “important” or “impactful” publications [3, 17]. While this emphasizes quality over quantity, it can create an opportunity for a new problem to arise in the form of a mismatch between the applicant’s and selection committee’s conceptions of what constitutes a high-quality paper.

With the rise of easy-to-access bibliometric data, a slew of metrics have been devised to enumerate quality in an “objective” way, the most notable being the Impact Factor [18]. The increased use of such metrics (in some cases far beyond their intended scope) to evaluate the quality of journals, papers, and individuals, has had consequences on the way journals and scientists operate. As a metric becomes more ubiquitous as a measure of quality, it tends to become a target, inspiring new approaches to “game” the system and achieve a higher rating [19, 20] (necessarily resulting in the metric’s loss of descriptive power [21]).

## Measuring the trade-off

Traditionally, scientists have sought to identify trade-offs between quantity and quality through comparing authors (or groups of authors) with high productivities to those with lower productivities, looking for correlation with a metric of quality. Yet, this approach poses a difficult problem of equivalence: how should one quantify the effect of age or specific field of study? Flexibility in identifying valid comparisons runs the risk of capturing undesired correlates, while a conservative approach results in small samples sizes and a lack of statistical power [22].

We take a different tack, comparing authors with themselves across time. If a trade-off between quantity and quality exists in scientific publishing, one would expect to find a negative relationship between the number of papers a scientist publishes in a given time-frame and the quality of those papers. Perhaps the simplest way to quantify this for a given author is to draw two of their papers at random and compare both their “quantity” and “quality”. If the paper with the higher quality is also the one coming from the less productive year, then there is evidence for a trade-off, and if it was published in the more productive year, there is evidence for a positive relationship. We can repeat this for all possible pairs of papers for a given author to determine whether or not (or how often) they experience a trade-off. Likewise, we can repeat this for very many authors to investigate the presence of a trade-off between quantity and quality more generally.

While quantity is relatively straightforward to enumerate as the number of papers published in the same year as a target paper, quality has proven to be a more controversial topic [8, 17, 20, 23]. The most prominent metrics for quality today rely, more or less directly, on the number of citations a given paper has accrued. This dependence on citations has been criticized [24, 25], but also demonstrated to correlate with other, independent metrics of quality [8, 26]. Despite their limitations, citations retain their popularity in part because the alternatives still lack the coverage, accuracy, and ease-of-use that citation counts maintain.

In this work, we use the number of citations as a proxy for quality, but find the results are also robust to using citation rates instead (S1 Supporting Information). In particular, because citation distributions have been observed to be log-normal [20], we take the log of the number of citations plus one. We analyze nearly 200,000 publications by more than 1600 members of the National Academy of Sciences between 1980 and 2006. Since all of the papers we consider in this analysis are at least ten years old, the confounding effects of paper aging and discovery should be reduced (repeating the analysis for alternative time-ranges yield similar results; S1 Supporting Information). While the choice of using members of the National Academy can be seen as a limitation, one advantage of this choice is that such authors are universally accepted as having made high-quality contributions, which provides a measure of confidence behind any relationship between quantity and quality we observe.

**Fig 1:**
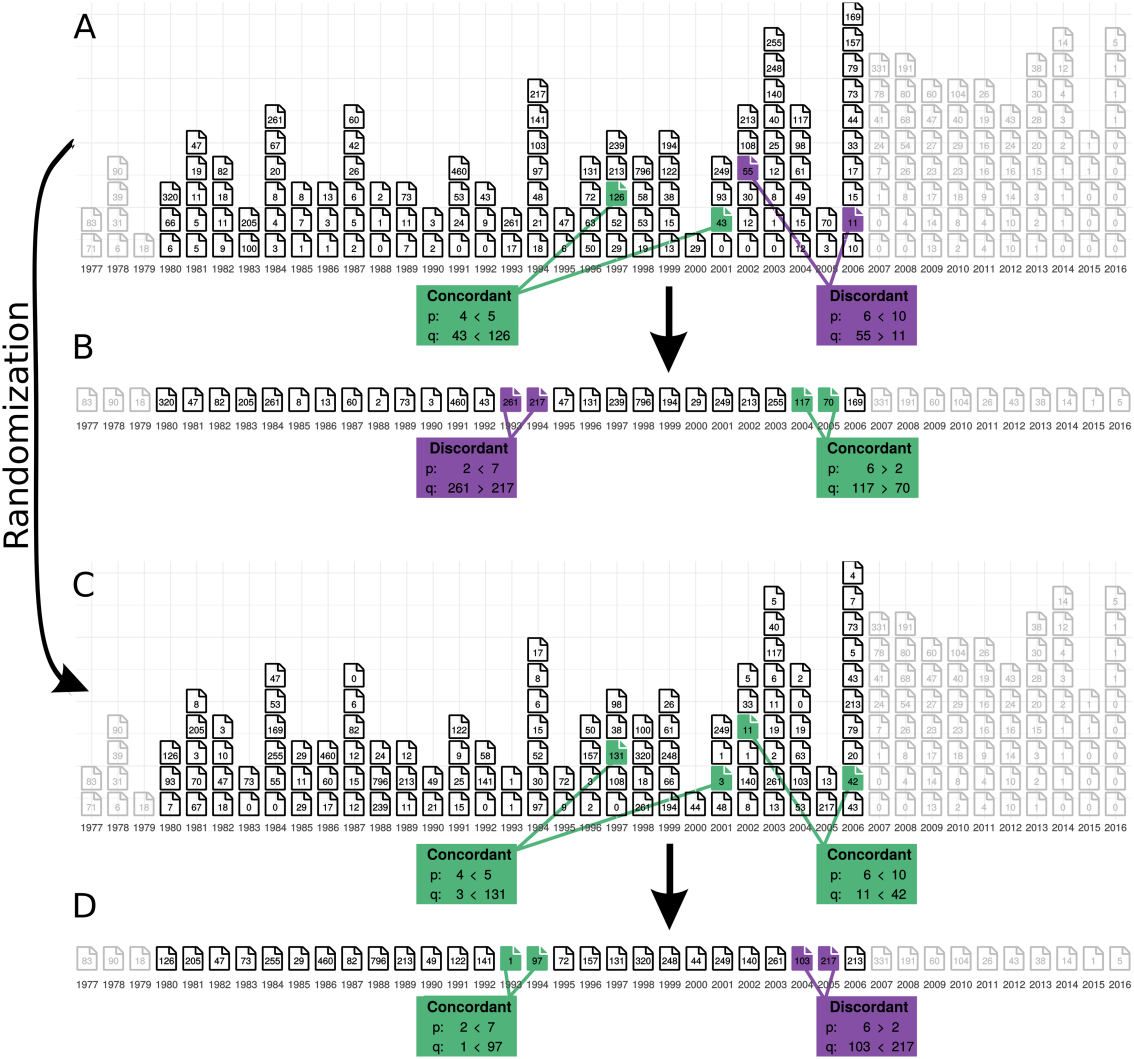
Sketch of the analysis. A scientist’s publication record can be depicted by the number of papers published each year and the number of citations each paper has accrued. Our analysis is restricted to those papers published between 1980 and 2006. To evaluate whether or not a scientist experiences a trade-off between quantity (the number of papers published in a given year, indicated by the number of stacked pages) and quality (shown here as the number of citations accrued by those papers for simplicity, and indicated by the number within each page icon), we take each pair of papers and compare the number of papers published in the same year with the number of citations each paper has (A). If the lower cited paper comes from the year with fewer publications, then we call the pair concordant, else we call it discordant. we then look at the number of pairs falling into each category and calculate a correlation coefficient (Eq 1). To reduce potential biases introduced by considering all possible pairings, we can select a summary statistic and only consider pairs of this statistic between adjacent years, *e*.*g*. the maximally-cited paper in each year (B). To get an understanding of our expectations for the number of concordant versus discordant pairs, we can take an empirical time-line and randomize the citation counts among an author’s publications and re-run the analysis (C-D). Note that choosing the same pairs in a randomized timeline can result in the same or different relationships between the *p*’s and *q*’s. For each author, we are interested in the proportion of all possible pairings that are concordant. Proportions less than 0.5 correspond to a *τ* less than 0 and are indicative of a trade-off between quantity and quality.

To quantify the relationship between quantity and quality, we take each possible pair of an author’s publications and compare both their quality *q* = *log*(#Citations + 1) (using the number of citations accumulated as of October 2016) and quantity, or productivity, *p*, defined as the number of papers published in the same year as the focal paper. Thus, each paper has an associated value for *p* and *q*. If *q*_1_ > *q*_2_ and *p*_1_ > *p*_2_ or *q*_1_ < *q*_2_ and *p*_1_ < *p*_2_, *i*.*e*. the paper with a higher number of citations was published in a year with higher quantity, then we call the pair concordant, while if the inequalities do not match up, *i*.*e*. the paper with a higher number of citations was published in the year with lower quantity, we call the pair discordant. Discordance is indicative of a trade-off (negative correlation) between these two values (Fig 1A).

We can then calculate a type of pairwise Kendall correlation, in which the correlation coefficient

$$\begin{array}{c} \tau =\frac{\#\text{Concordant}-\#\text{Discordant}}{\#\text{Total pairs}}. \end{array}$$ (1)

**Fig 2:**
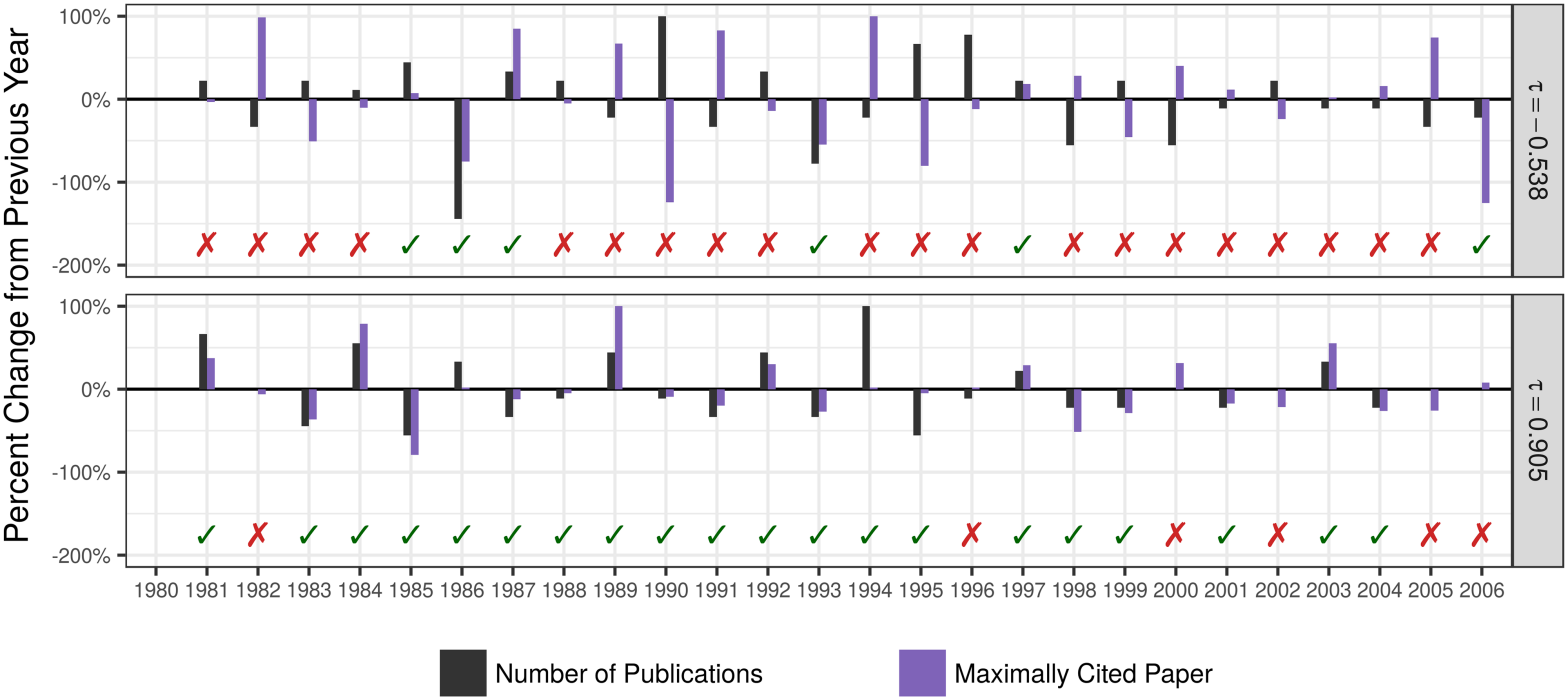
Example trajectories of scientists who experience a trade-off or concordance. The percent change in productivity (black) and quality (specifically the number of citations received by the maximally cited paper in that year; purple) from the previous year. Years in which the two changes are both of the same sign are termed concordant (green check-mark), while years in which the sign of the change differs are termed discordant (red X). The top scientist experiences a strong trade-off (low *τ*, many discordant years), while the bottom scientist experiences strong concordance between quantity and quality.

Thus, −1 ≤ *τ* ≤ 1, where a value of −1 indicates that, for every pair, the higher quality paper was published in the year with lower quantity (*i*.*e*. there is a perfect trade-off between quantity and quality) and a value of 1 indicates that, for every pair, the higher quality paper was published in the year with higher quantity (*i*.*e*. there is perfect concordance between quality and quantity) (Fig 2).

There are two issues with this naive analysis. First, it ignores the effect of time on the researcher herself: as a researcher produces more papers, they grow more experienced, their laboratory develops, their name becomes more established, and possibly their research topics evolve as well. To address this, we implement a restriction to the above analysis, in which we introduce a temporal “sliding window”: only comparing papers published within 1, 2, 3, … years of each other (results for windows greater than 1 are presented in S1 Supporting Information). Second, and more subtly, it introduces a bias in the form of a variable number of possible pairwise comparisons between years: more productive years have more possible pairings than do less productive years. To address this, we can collapse the distribution of citation counts in a given year to a single summary statistic, consolidating quality to just one value per year (Fig 1B). This also allows the expansion of our analysis, since we can choose any statistic for the comparison. In our analysis, we look at the effect of productivity on the median, mean, maximum, and minimum number of citations for each year.

## Results and discussion

**Fig 3:**
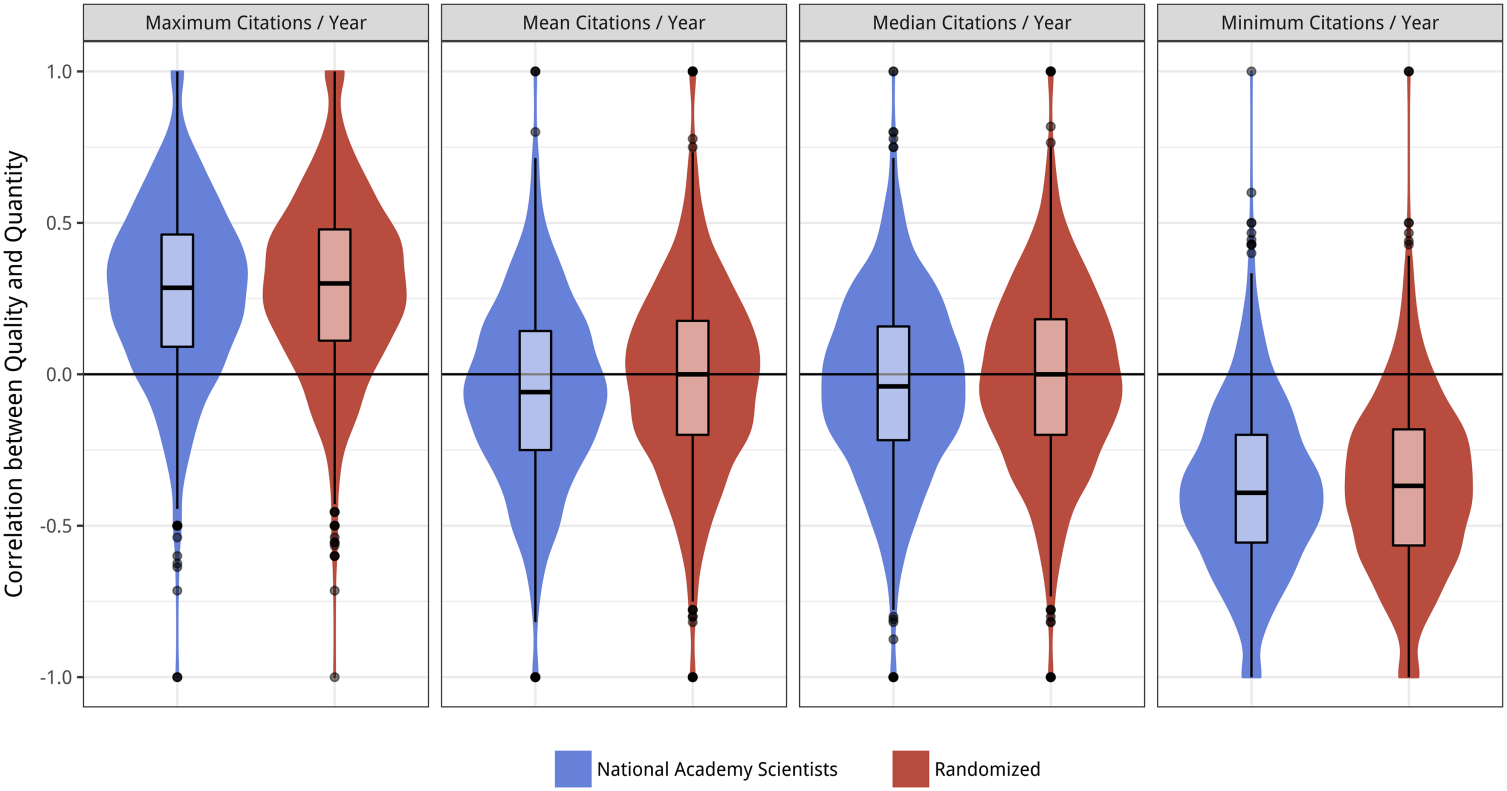
Violin plots of the strength of pairwise correlation between quantity and quality for members of the National Academy of Sciences. The panels are divided based on which summary statistic is being compared across years (*e*.*g*. maximally-cited paper published in that year). A value of 1 (-1) indicates that, for every pair of adjacent years, the more productive one had a higher (lower) statistic. A value of 0 (horizontal black line) indicates that the larger statistic is equally likely to be from the more or less productive year. In blue (left in each plot) are the empirically observed correlation values for each author. In red (right in each plot) are the correlation values observed when citation counts were randomized within each author’s corpus.

Quantifying productivity as the number of papers published in a given year and quality as the citation count of the maximally-cited paper from that year, we find that there is a positive relationship between the two: the most cited paper in years of greater productivity more often than not garners more citations than does the maximum in less productive years. Yet, if one looks at the minimally-cited paper, the opposite trend is observed: higher numbers of citations are observed in years of lower productivity (Fig 3). Thus, for a given scientist, more productive years yield both lower minimum and higher maximum quality papers. As for the central tendencies (mean and median), we see no effect of quantity on quality: the higher mean (median) number of citations for a given pair of years is equally likely to come from the more productive as the less productive year.

### Null expectations

**Fig 4:**
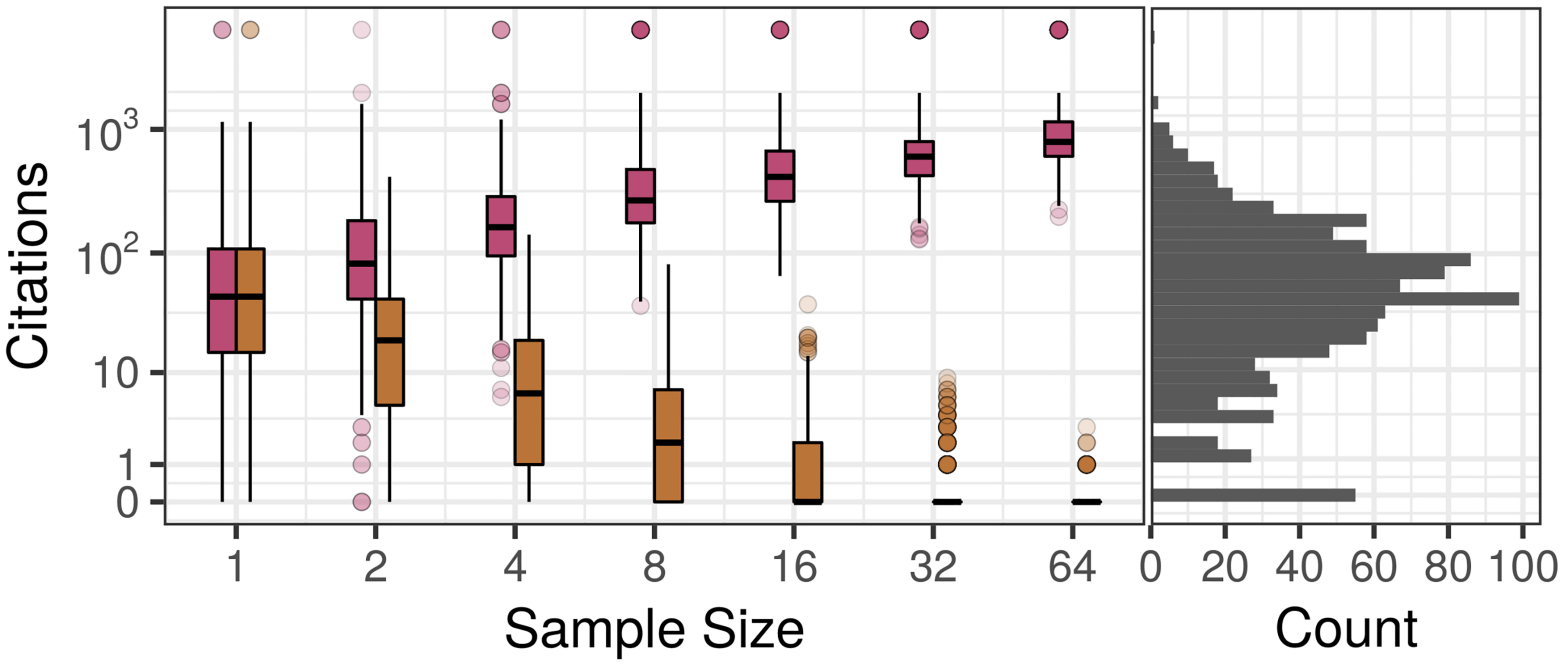
The relationship between sample size and the maximum/minimum citation count drawn in that sample. Right: histogram of citation counts for a given member of the National Academy of Sciences across their more than 1000 publications. Left: boxplots for the maximum (red, top) and minimum (orange, bottom) citation count drawn in samples of the size indicated by the horizontal axis. Each sample of a given size was repeated 1000 times to generate the distributions indicated by the boxplots.

The direction of these trends is consistent with the expectations for increasing sample size when drawing randomly from a distribution (Fig 4), prompting the question of what kind of distribution to expect if there were no relationship between quality and quantity. Is this result evidence of an interaction between quality or quantity, or simply a product of the underlying distributions of citations and productivities? Furthermore, we would like to quantify how well a null hypothesis of random sampling can explain the magnitudes of the observed discrepancies from *τ* = 0.

We can investigate this explicitly by repeating the analysis with an additional step of (prior to performing the pairwise comparisons) scrambling the citation counts among an author’s publications (Fig 1C), effectively breaking any mechanistic relationship between quantity and quantity. We find that the randomized data produces nearly the same distributions as observed for the empirical data. This suggests that most of the effects above can be explained by the underlying distribution of citations, without having to invoke any mechanistic relationship between producing more papers and producing better ones. Put another way, a scientist’s best paper is better in a more productive year because they are getting more draws from the possible citation counts, and their worst paper is worse for the same reason.

This result, though broader in scope, agrees with other studies on the distribution of impact throughout one’s scientific career. For instance, Sinatra *et al*. [27] recently published findings on what they call the “random-impact rule” wherein the likelihood of publishing one’s most successful paper is constant throughout one’s publishing career (after controlling for the changing productivity over time). Likewise, Moreira *et al*. [20] found that the asymptotic number of citations an author or institution is likely to accumulate can be consistently approximated using a discrete log-normal distribution. Finally and much earlier, Simonton [28] elucidated his “chance-configuration theory” in which the key difference between the genius and the non-genius is “the cognitive and motivational capacity to spew forth a profusion of chance permutations pertaining to a particular problem.”

### The subtle trade-off

Though the majority of the observed effect can be explained by random sampling, there is still a small but statistically significant deviation of the empirical correlations from the randomly sampled ones in most cases (Table 1). Furthermore, in almost all of these cases, the empirical mean is lower than that observed for the random values—re-igniting the possibility of a trade-off between quality and quantity in scientific publishing. That is, though scientists who publish more tend to also get more citations on their highest cited papers, they get fewer citations than would be expected if citations were assigned by random sampling. This subtle trade-off was present for mean and median citation counts as well, with only the minimally cited paper (which has a natural lower bound of 0 Citations) not following the trend.

**Table 1:** Kolmogorov-Smirnov test for differences between randomized and empirical distributions.

	Citation Distribution Summary Statistic			
Window Size	Maximum	Mean	Median	Minimum
1	0.04	0.08***	0.04	0.05
2	0.07***	0.07***	0.08***	0.02
3	0.09***	0.09***	0.08***	0.03
4	0.09***	0.07***	0.07***	0.02
5	0.09***	0.07***	0.08***	0.05*
Inf	0.06**	0.10***	0.09***	0.17***

Entries are structured as [statistic]^[significance]^, where the statistic is the Kolmogorov-Smirnov test statistic and significance is indicated by *, **, and *** to signify *p* < 0.05, *p* < 0.01, and *p* < 0.001, respectively.

## Further considerations

### Growth in science

A concern that often emerges in discussions of scientific quantity and quality is the striking rise in the number of scientific publications over the past decades [2, 6, 29], or, more specifically, the increase in per capita publications. We see this in our data as well, though not quite as dramatically, with members of the National Academy of Sciences (NAS) publishing, on average, 0.084 (*p* ≪ 0.001) more papers-per-year per year over the past 75 years. Put another way, the average output of a member of the NAS increases by about one paper-per-year every twelve years (S1 Supporting Information).

This rise has been attributed to reward and evaluation systems, co-authorship, and attempts to optimize with respect to bibliometrics [30]. Individual scientists’ careers often depend on measures of quantity and quality [14], inspiring research practices, such as the “Least Publishable Unit”, and gratuitous co-authorship, which have been popular harbingers of the decline of quality in the pursuit of quantity [3, 30].

### Multiple authorship

This analysis does not take the issue of multiple authorship into account. Though the large sample size should alleviate the risk of idiosyncrasies, it is not infeasible that in years that authors collaborate more, they are also able to produce more papers and the relationship between author number and citation count is well known [8, 23]. This is a more mechanistic explanation than the random sampling, but we do not have the information required to test that hypothesis in the present study. Nevertheless, the accuracy and simplicity of the random sampling hypothesis are encouraging. Furthermore, since we only consider adjacent years in our analysis and are comparing authors with themselves, we do not expect the level of co-authorship to vary widely within any given pairwise comparison, suggesting that the magnitude of this effect would likely be small if it is indeed present.

### Self-citation

Self-citation is a common critique and significant issue when using citation counts as a measure of quality [31–33]. Since we compare authors with themselves, this could be a concern if self-citing practices co-varied with publication rates, *e*.*g*. if an author more frequently self-cites her most-cited paper from more productive years than the corresponding most-cited paper from less productive ones. We can not investigate this possibility directly with the data we have collected, yet we do not believe this to be the case for the following reasons.

First, we do not see an incentive for authors to selectively cite papers from more productive years than less productive ones. Indeed, if one’s aim were to “game” the system, as alluded to above, a self-citation strategy might differ depending on the metric. For at least some such metrics in use today, *e*.*g*. the h- or g-indexes, (assuming self-citation-corrected calculations are not used to begin with) the best strategy would not be to produce one, highly cited paper, but rather, to spread self-citations among many papers to get more papers above a given citation threshold. Second, most metrics do not take year of publication into account, so there is not an obvious incentive for the papers an author choses to inflate coming from more, as opposed to less productive years. Third, the percentage of citations for a given work that are self-citations has been observed decreases over time [34], so we expect this effect to be minimized by our consideration of papers which are at least a decade old. Finally, if the papers being self-cited are indeed the high-quality ones [8], then this form of self-citation is not manipulative, but rather an honest signal of quality (but see [35]).

### Alternative explanations

A mechanistic alternative to the hypothesis of random sampling is that each scientist has some inherent ability and our measures of quality and quantity are actually just a reflection of this hidden variable. Alternatively, there could be a positive feedback loop operating in which the acts of publishing and receiving citations increase one’s propensity of producing more papers and receiving more citations. Allison and Stewart [36] describe these two hypotheses as the “sacred spark” and “accumulative advantage.” The latter has also been referred to as a manifestation of the “Matthew Effect”, [37]. Our study does not preclude these possibilities, as each author is being compared to themselves and the “random draws” are being taken from the author’s own distribution of citation counts. Differences between different authors quality distributions can, and likely do, exist [27].

To investigate these hypotheses more thoroughly in the framework presented here, we would have to re-structure the hypotheses. For instance, for accumulative advantage to explain these results, it would have to be temporally restricted: in years that a scientist is most inspired, he produces more papers which gain him reputation and thus more citations. Yet, this recognition would have to fade rapidly, such that the gained citations only apply to papers published in that same year. Thus, while we cannot conclusively exclude these mechanisms from the interpretation of our results, our method of comparing authors with themselves and only across adjacent years substantially reduces the intuitiveness of these explanations.

## Conclusions

Regardless of the underlying mechanisms at play which might produce the observed empirical pattern (*e*.*g*. multiple authorship, self-citation, *etc*.), the fact that this pattern is so closely replicated by completely scrambling an authors citation record suggest that such explanations are not *necessary* to explain these results. Even if all of these mechanisms are nullified by completely scrambling citation counts among an author’s publications, we still see increased maximum citations and decreased minimum citations in more productive years.

Yet, perhaps the most interesting result is the observed “second-order effect” of the subtle trade-off. This consistent discrepancy between the empirical and randomized data suggest the existence of a small, but statistically significant, negative relationship between quantity and quality. This slight deviation from random expectation suggests an inroad for the role for a more mechanistic explanation. Many authors have proposed explanations for such a trade-off, but further research needs to be done to demonstrate and classify these potential mechanisms.

It is clear that many challenges have arisen as a result of the rapid growth of science and scientific literature. For example, it has become impossible to keep abreast of all publications that might be relevant or instructive for one’s research program, and as a consequence, we have seen a rise in co-authorship and specialization [5]. It is tempting, especially for scientists, to take these observations and try to identify trends and explanations. Yet rarely are these explanations approached with the same rigor scientists apply to their primary research. There is a dearth of statistical appraisal of how science works (and doesn’t), and the studies that are conducted often reveal unanticipated results. With respect to this study, it is an open question whether these results generalize to the greater scientific community (beyond the National Academy) and if/how they differ between specialties.

These results are not proof for a causal relationship. If a scientist increases their publication rate, they will not necessarily produce their best paper ever. In fact, while scientists tend to produce better best-papers in the years that they publish more, they also produce more low-quality papers and worse worst-papers. It turns out the trade-off in scientific publishing is more nuanced than previously suggested. Importantly, if citation accumulation is indeed dominated by stochasticity, systems of incentives and rewards based on citations should be re-examined. Scientists tend to think of science as a meritocratic enterprise, but this study provides another piece to a growing body of work calling that assumption into question.

## Materials and methods

For each member of the National Academy of Sciences in one of twenty-one Primary Sections we attempted to extract all affiliated ScopusIDs. We searched the Scopus Abstract and Citation Database for each ScopusID to identify all corresponding published articles. For each paper we downloaded a unique identifier for the paper, the year of publication, and the number of citations it has received through October 2016. We were able to thus identify 1966 (97%) of the 2,027 members of the Academy within the selected sections (1729 (85%) of which had at least one article cataloged on Scopus), and extract information about their collective 320,294 published papers.

We restricted our analysis to papers published between 1980 and 2006 (inclusive). This time-range balanced the availability of accurate citation records with consideration of the effects of paper age and discovery. Finally, we restricted the analysis to authors with at least twenty publications in this time-frame. This was to ensure sufficient potential pairs to evaluate the correlation. Following these constraints brought our final sample size to 1629 authors and 194,952 publications.

Code to replicate the analysis and data collection are available online at https://git.io/vMPoB

## Supporting information

S1 Supporting Information Repeated analyses for additional quality metrics and time-ranges and other supporting information. (PDF)

## References

[1] Rubin Z. On Measuring Productivity by the Length on One’s Vita. Personality and Social Psychology Bulletin. 1978;4(2):197–198. 10.1177/014616727800400203

[2] Fischer J, Ritchie EG, Hanspach J. Academia’s obsession with quantity. Trends in ecology & evolution. 2012;27(9):473–474. 10.1016/j.tree.2012.05.010 22727015

[3] Halme P, Komonen A, Huitu O. Solutions to replace quantity with quality in science. Trends in Ecology & Evolution. 2012;27(11):586 10.1016/j.tree.2012.08.007 22959279

[4] Haslam N, Laham SM. Quality, quantity, and impact in academic publication. European Journal of Social Psychology. 2010;40(2):216–220.

[5] Donaldson MR, Cooke SJ. Scientific publications: Moving beyond quality and quantity toward influence. BioScience. 2013; p. bit007.

[6] Sarewitz D. The pressure to publish pushes down quality. Nature. 2016;533(7602):147–147. 10.1038/533147a 27172010

[7] Dennis W. Bibliographies of eminent scientists. The Scientific Monthly. 1954;79(3):180–183.

[8] Lawani SM. Some bibliometric correlates of quality in scientific research. Scientometrics. 1986;9(1–2):13–25. 10.1007/BF02016604

[9] Abramo G, D’Angelo CA, Costa FD. Testing the trade-off between productivity and quality in research activities. Journal of the American Society for Information Science and Technology. 2010;61(1):132–140. 10.1002/asi.21254

[10] Huang Dw. Positive correlation between quality and quantity in academic journals. Journal of Informetrics. 2016;10(2):329–335. 10.1016/j.joi.2016.02.002

[11] Cole S, Cole JR. Scientific output and recognition: A study in the operation of the reward system in science. American sociological review. 1967; p. 377–390. 10.2307/2091085 6046811

[12] Loyola RD, Diniz-Filho JAF, Bini LM. Obsession with quantity: a view from the south. Trends in Ecology & Evolution. 2012;27(11):585 10.1016/j.tree.2012.07.016 22917847

[13] Kelchtermans S, Veugelers R. The great divide in scientific productivity: Why the average scientist does not exist. Industrial and Corporate Change. 2011;20(1):295–336. 10.1093/icc/dtq074

[14] Feist GJ. Quantity, quality, and depth of research as influences on scientific eminence: Is quantity most important? Creativity Research Journal. 1997;10(4):325–335.

[15] Paulus FM, Rademacher L, Schäfer TAJ, Müller-Pinzler L, Krach S. Journal impact factor shapes scientists’ reward signal in the prospect of publication. PloS one. 2015;10(11):e0142537 10.1371/journal.pone.0142537 26555725 PMC4640843

[16] Alberts B. Impact factor distortions. Science. 2013;340(6134):787–787. 10.1126/science.1240319 23687012

[17] Benedictus R, Miedema F, Ferguson M. Fewer numbers, better science. Nature. 2016;538(7626):453 10.1038/538453a 27786219

[18] Garfield E. Citation Indexes for Science: A New Dimension in Documentation through Association of Ideas. Science. 1955;122(3159):108–111. 10.1126/science.122.3159.108 14385826

[19] Editors TPM. The Impact Factor Game. PLOS Medicine. 2006;3(6). 10.1371/journal.pmed.0030291 PMC1475651 16749869

[20] Moreira JA, Zeng XHT, Amaral LAN. The Distribution of the Asymptotic Number of Citations to Sets of Publications by a Researcher or from an Academic Department Are Consistent with a Discrete Lognormal Model. PLOS one. 2015;10(11):e0143108 10.1371/journal.pone.0143108 26571133 PMC4646658

[21] Goodhart CA. Problems of monetary management: the UK experience In: Monetary Theory and Practice: The UK Experience. Springer; 1984 p. 91–121.

[22] Kaur J, Ferrara E, Menczer F, Flammini A, Radicchi F. Quality versus quantity in scientific impact. Journal of Informetrics. 2015;9(4):800–808. 10.1016/j.joi.2015.07.008

[23] Lindsey D. Production and citation measures in the sociology of science: The problem of multiple authorship. Social Studies of Science. 1980;10(2):145–162. 10.1177/030631278001000202

[24] Lindsey D. Using citation counts as a measure of quality in science measuring what’s measurable rather than what’s valid. Scientometrics. 1989;15(3–4):189–203. 10.1007/BF02017198

[25] Kivimäki M, Batty GD, Kawachi I, Virtanen M, Singh-Manoux A, Brunner EJ. Don’t let the truth get in the way of a good story: an illustration of citation bias in epidemiologic research. American journal of epidemiology. 2014; p. kwu164. 10.1093/aje/kwu164 PMC4128774 24989242

[26] Cole J, Cole S. Measuring the Quality of Sociological Research: Problems in the Use of the “Science Citation Index”. The American Sociologist. 1971;6:23–29.

[27] Sinatra R, Wang D, Deville P, Song C, Barabási AL. Quantifying the evolution of individual scientific impact. Science. 2016;354(6312):aaf5239 10.1126/science.aaf5239 27811240

[28] Simonton DK. Chance-configuration theory of scientific creativity. The psychology of science: Contributions to metascience. 1989; p. 170–213.

[29] Price DJdS. Little Science, Big Science. New York: Columbia University Press; 1963.

[30] Broad WJ. The publishing game: getting more for less. Science. 1981;211(4487):1137–1139. 10.1126/science.7008199 7008199

[31] MacRoberts MH, MacRoberts BR. Problems of citation analysis: A critical review. Journal of the American Society for information Science. 1989;40(5):342 10.1002/(SICI)1097-4571(198909)40:5<342::AID-ASI7>3.0.CO;2-U

[32] Seglen PO. Citations and journal impact factors: questionable indicators of research quality. Allergy. 1997;52(11):1050–1056. 10.1111/j.1398-9995.1997.tb00175.x 9404555

[33] Fowler JH, Aksnes DW. Does self-citation pay? Scientometrics. 2007;72(3):427–437. 10.1007/s11192-007-1777-2

[34] Rousseau R. Temporal differences in self-citation rates of scientific journals. Scientometrics. 1999;44(3):521–531. 10.1007/BF02458493

[35] Gami AS, Montori VM, Wilczynski NL, Haynes RB. Author self-citation in the diabetes literature. Canadian Medical Association Journal. 2004;170(13):1925–1927. 10.1503/cmaj.1031879 15210641 PMC421720

[36] Allison PD, Stewart JA. Productivity differences among scientists: Evidence for accumulative advantage. American sociological review. 1974; p. 596–606. 10.2307/2094424

[37] Merton RK. The Matthew effect in science. Science. 1968;159(3810):56–63. 10.1126/science.159.3810.56 5634379

