# Supplementary material for: And, not or: Quality, quantity in scientific publishing
Source: PLoS One. 2017 Jun 1;12(6):e0178074. doi: 10.1371/journal.pone.0178074 (PMC5453441; doi:10.1371/journal.pone.0178074)
Supplement: S1 Supporting Information — (PDF) [file pone.0178074.s001.pdf]

Supplemental Materials for: And, not Or: Quality, Quantity in Scientific Publishing

Matthew J. Michalska-Smith & Stefano Allesina

March 20, 2017

These supplemental materials contain repeated and extended analyses to those found in the main text to demonstrate the generality of the results and their robustness to fundamental assumptions, such as the chosen metric of quality and the time-frame being analyzed. Sections 1 to 3 show qualitatively similar results upon varying the metric used to quantify quality, Section 4 considers an alternative time-frame, and Section 5 shows the general trend of productivity through time.

Contents

1 Log Citation Count 1980-2006 **2**

2 Citation Rate 1980-2006 **7**

3 Raw Citation Count 1980-2006 **10**

4 Log Citation Count 2000-2015 **13**

5 Productivity Through Time 1948-2015 **16**

1 Log Citation Count 1980-2006

Tables

Table S1: Student's t-Test comparing means of Randomized to Empirical distributions of concordance values using log citation counts (1980-2006).

nyears	All	Maximum	Mean	Median	Minimum
1	0.01**	0.01*	0.02***	0.01*	0.01
2	0.01***	0.02***	0.02***	0.02***	0.00
3	0.01***	0.02***	0.02***	0.02***	0.00
4	0.01***	0.02***	0.02***	0.01***	0.00
5	0.01***	0.02***	0.02***	0.02***	-0.01*
Inf	0.00*	0.01***	-0.01***	-0.01***	-0.04***

Entries are structured as [statistic]$^{\text{[significance]}}$, where the statistic is Student's t and significance is indicated by $*$, $**$, and $***$ to signify $p < 0.05$, $p < 0.01$, and $p < 0.001$, respectively. A positive value indicates that the randomized distribution has a higher mean than the empirical distribution, *i.e.* higher productivity is associated with higher citation rates more often in the randomized records than in the empirically observed ones.

Table S2: Kolmogorov-Smirnov Test comparing Randomized to Empirical distributions of concordance values using log citation counts (1980-2006).

Transformation	nyears	All	Maximum	Mean	Median	Minimum
none	1	0.08***	0.04	0.08***	0.04	0.05
	2	0.09***	0.07***	0.07***	0.08***	0.02
	3	0.10***	0.09***	0.09***	0.08***	0.03
	4	0.08***	0.09***	0.07***	0.07***	0.02
	5	0.09***	0.09***	0.07***	0.08***	0.05*
	Inf	0.11***	0.06**	0.10***	0.09***	0.17***
shifted mean	1	0.03	0.04	0.07***	0.06*	0.04
	2	0.04	0.03	0.04	0.04	0.04
	3	0.04	0.03	0.04	0.03	0.04
	4	0.04	0.03	0.02	0.03	0.02
	5	0.05*	0.02	0.02	0.02	0.02
	Inf	0.09***	0.04	0.06**	0.05*	0.06*
mean 0, sd 1	1	0.02	0.04	0.06**	0.06*	0.04
	2	0.04	0.03	0.04	0.04	0.04
	3	0.03	0.03	0.04	0.03	0.04
	4	0.04	0.03	0.02	0.03	0.02
	5	0.03	0.02	0.03	0.02	0.03
	Inf	0.03	0.02	0.03	0.03	0.03

Entries are structured as [statistic]$^{\text{[significance]}}$, where the statistic is the Kolmogorov-Smirnov test statistic and significance is indicated by $*$, $**$, and $***$ to signify $p < 0.05$, $p < 0.01$, and $p < 0.001$, respectively. The distribution of concordance values being compared is generated from all possible pairings of papers published within "nyears" of each other ("All") or just the "Maximum", "Mean", "Median", or "Minimum" citation rate in each year being compared between years. The distributions being compared are untransformed ("none"), have the randomization means shifted according to the difference identified by a Student's t-Test (see Table S1), or have both distributions rescaled to have means of 0 and standard deviations of 1.

Figure Captions

Figure S1 Similar to Figure 1 in the main text, but for all sliding window sizes and for all possible pairings of papers as well as pairings of summary statistics. Colored lines in box-plots indicate the location of the median of the complementary box-plot for ease of comparison. Note that a sliding window of infinite width ("Inf") is equivalent to comparing all possible pairs, regardless of temporal distance. The "All Pairs" column refers to running the analysis on all possible pairs of papers with no reduction via summary statistics.

Figure S2 Trends in the significance level of Kolmogorov-Smirnov Test statistics with increasing sliding window size (*i.e.* another way to view the information presented in Table S2). Color/shape correspond to the transformation (if any) applied to the data prior to analysis. The facets indicate which pairs are considered (all possible pairs or just one "representative" value per year). Points below the horizontal black line are insignificant (p-value > 0.05). The rapid decline of significance with shifting of the mean and re-scaling suggest the bulk of the difference observed in the distributions is due to a difference in mean, rather than a difference in shape.

Figure S3 Distribution of author p-values when compared to the distribution of 1000 randomizations of their Citations throughout their publication record. The horizontal line indicates the expected density of authors at any given p-value. For this figure, authors are restricted to those having at least 100 publications in the time-frame, rather than 20 as in other figures and statistics.

Figures

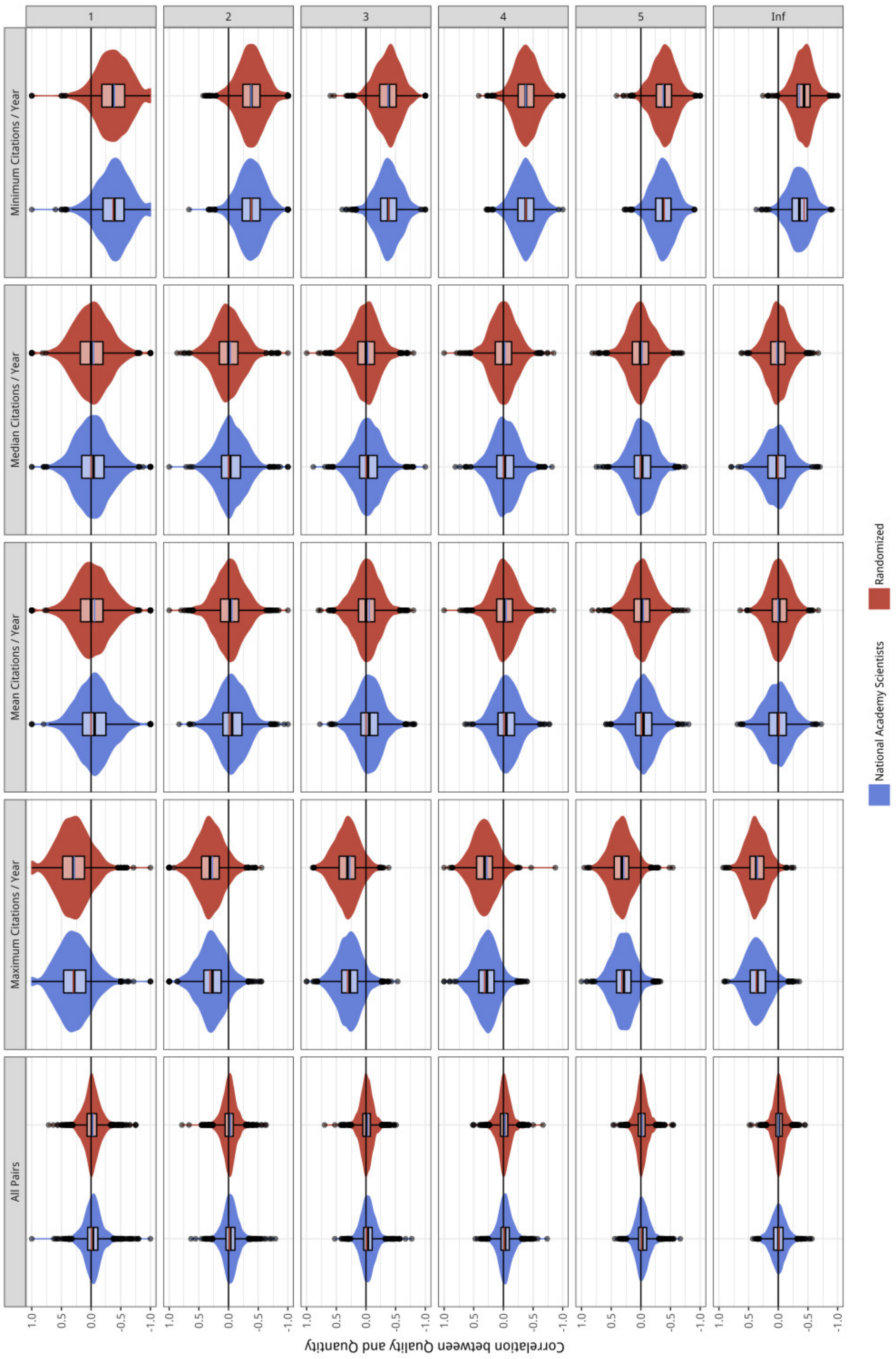

Figure S1: See caption above.

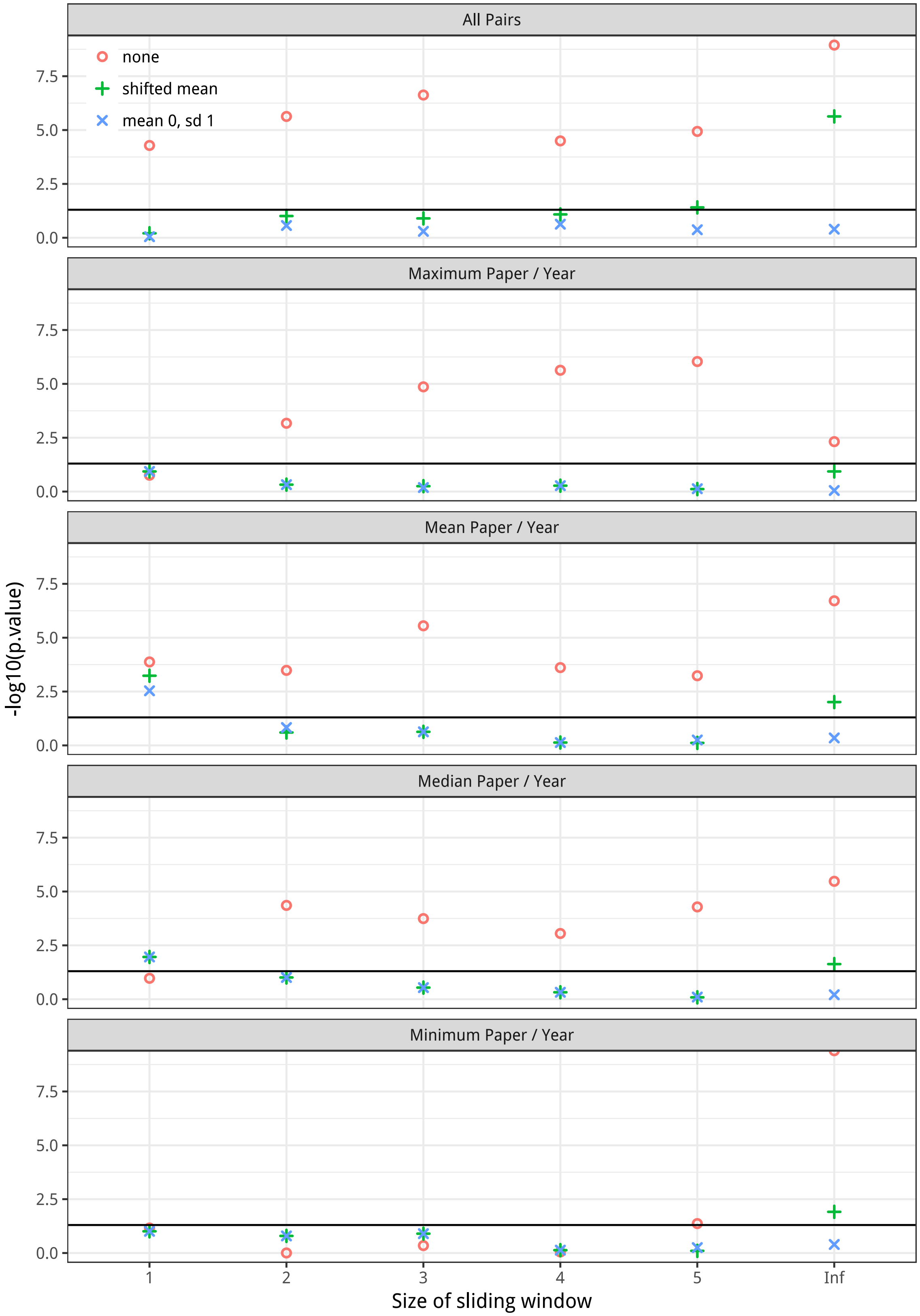

Figure S2: See caption above.

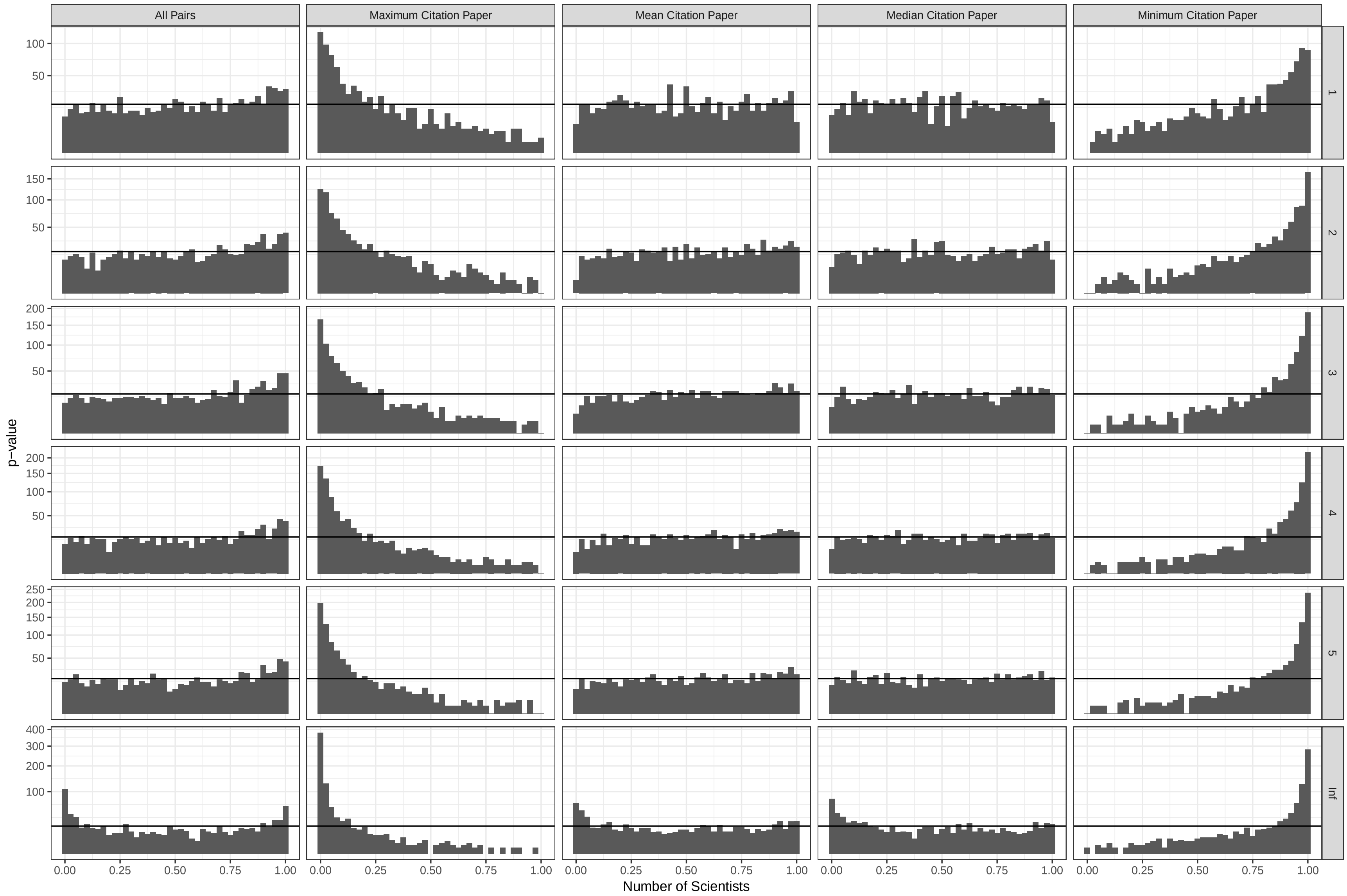

Figure S3: See caption above.

2 Citation Rate 1980-2006

Table S3: Student's t-Test comparing means of Randomized to Empirical distributions of concordance values using citation rate (1980-2006).

nyears	All	Maximum	Mean	Median	Minimum
1	0.01***	0.02***	0.03***	0.02***	0.01
2	0.01***	0.02***	0.03***	0.02***	0.00
3	0.01***	0.02***	0.02***	0.02***	0.00
4	0.01***	0.01***	0.02***	0.01**	-0.01
5	0.01***	0.01*	0.01**	0.00	-0.01**
Inf	-0.02***	0.00	-0.02***	-0.04***	-0.05***

Entries are structured as [statistic]$^{\text{[significance]}}$, where the statistic is Student's t and significance is indicated by $*$, $**$, and $***$ to signify $p < 0.05$, $p < 0.01$, and $p < 0.001$, respectively. A positive value indicates that the randomized distribution has a higher mean than the empirical distribution, *i.e.* higher productivity is associated with higher citation rates more often in the randomized records than in the empirically observed ones.

Table S4: Kolmogorov-Smirnov Test comparing Randomized to Empirical distributions of concordance values using citation rate(1980-2006).

Transformation	nyears	All	Maximum	Mean	Median	Minimum
none	1	0.09***	0.09***	0.10***	0.05*	0.03
	2	0.08***	0.09***	0.12***	0.08***	0.02
	3	0.09***	0.08***	0.10***	0.07***	0.02
	4	0.08***	0.08***	0.09***	0.05*	0.03
	5	0.07***	0.05*	0.06**	0.04	0.07**
	Inf	0.23***	0.07**	0.12***	0.18***	0.23***
shifted mean	1	0.03	0.05*	0.04	0.04	0.04
	2	0.03	0.04	0.04	0.05*	0.03
	3	0.04	0.03	0.04	0.03	0.03
	4	0.05*	0.03	0.03	0.02	0.02
	5	0.06**	0.02	0.02	0.03	0.02
	Inf	0.13***	0.07**	0.07***	0.09***	0.06**
mean 0, sd 1	1	0.02	0.05*	0.05*	0.04	0.04
	2	0.02	0.04	0.04	0.05*	0.03
	3	0.02	0.03	0.04	0.03	0.02
	4	0.03	0.03	0.03	0.02	0.02
	5	0.06*	0.02	0.02	0.03	0.02
	Inf	0.05	0.02	0.03	0.02	0.02

Entries are structured as [statistic]$^{\text{[significance]}}$, where the statistic is the Kolmogorov-Smirnov test statistic and significance is indicated by $*$, $**$, and $***$ to signify $p < 0.05$, $p < 0.01$, and $p < 0.001$, respectively. The distribution of concordance values being compared is generated from all possible pairings of papers published within "nyears" of each other ("All") or just the "Maximum", "Mean", "Median", or "Minimum" citation rate in each year being compared between years. The distributions being compared are untransformed ("none"), have the randomization means shifted according to the difference identified by a Student's t-Test (see Table S3), or have both distributions rescaled to have means of 0 and standard deviations of 1.

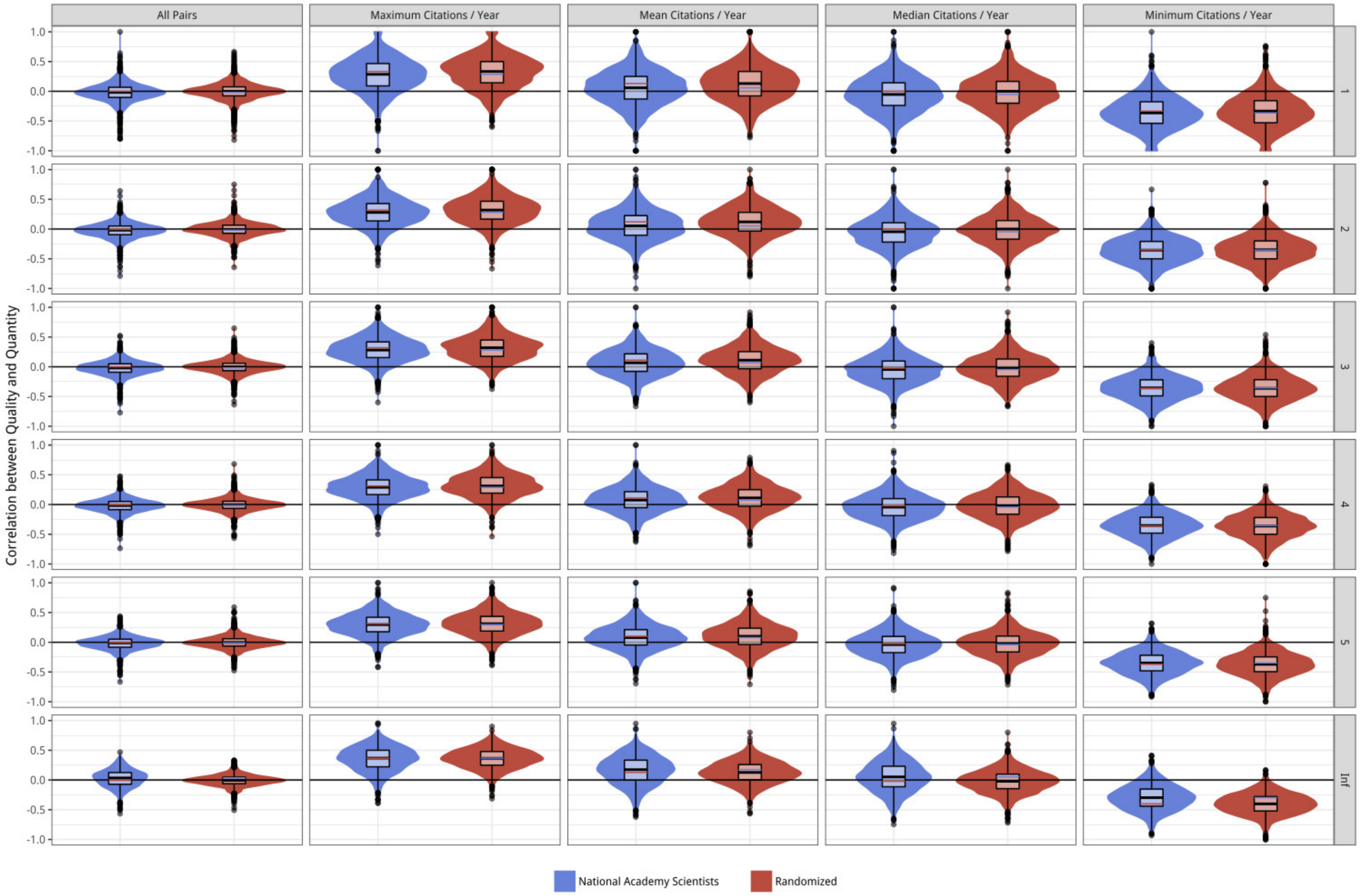

Figure S4: As Fig. S1, but using citation rates (#citations/years since publication) rather than log citations as the metric of quality. Colored lines in box-plots indicate the location of the median of the complementary box-plot for ease of comparison. Note that a sliding window of infinite width ("Inf") is equivalent to comparing all possible pairs, regardless of temporal distance. The "All Pairs" column refers to running the analysis on all possible pairs of papers with no reduction via summary statistics.

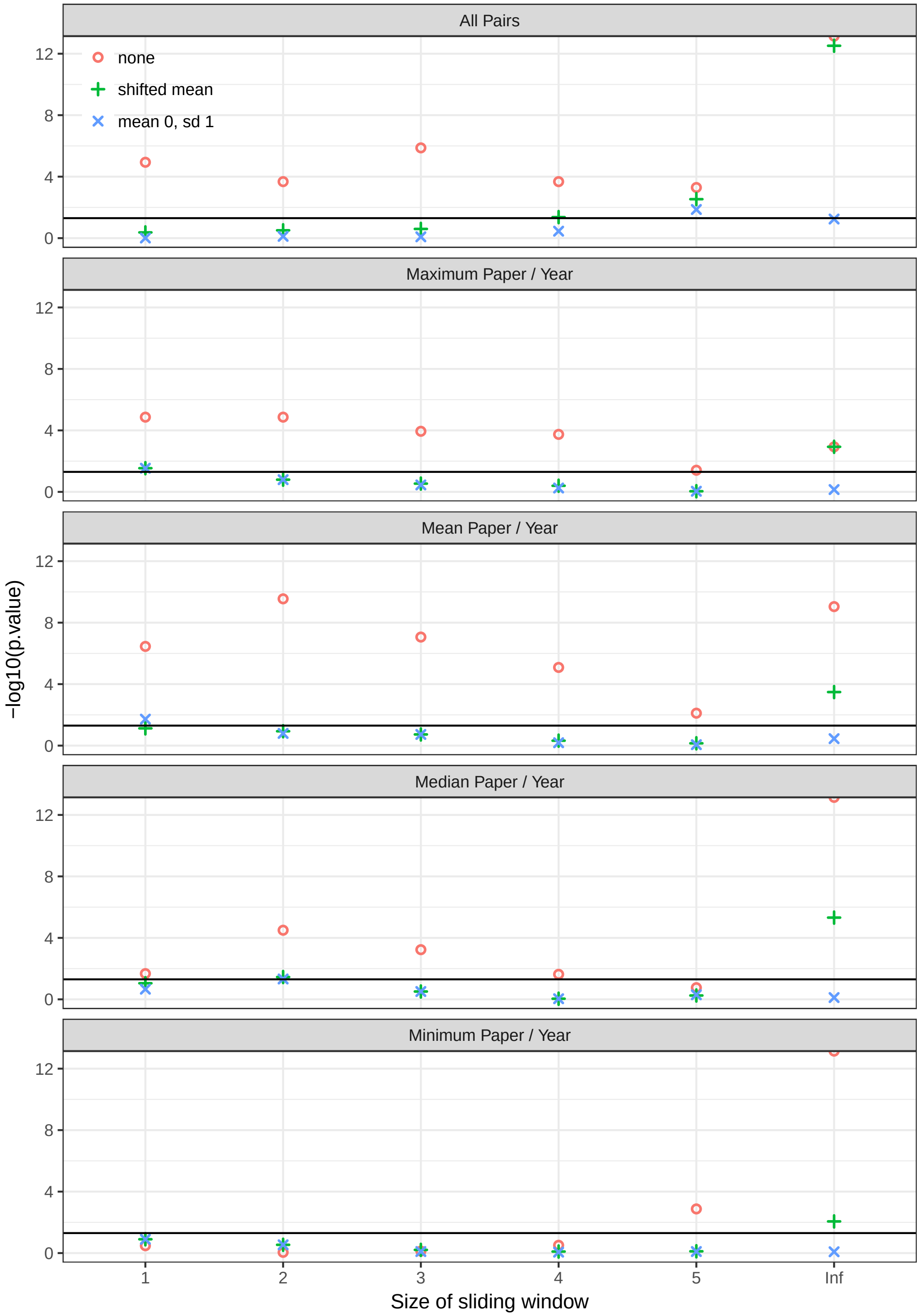

Figure S5: As Fig. S2, but using citation rates (#citations/years since publication), rather than log citations as the metric of quality. Trends in the significance level of Kolmogorov-Smirnov Test statistics with increasing sliding window size. Color/shape correspond to the transformation (if any) applied to the data prior to analysis. The facets indicate which pairs are considered (all possible pairs or just one "representative" value per year). Points below the horizontal black line are insignificant (p-value > 0.05). The rapid decline of significance with shifting of the mean and re-scaling suggest the bulk of the difference observed in the distributions is due to a difference in mean, rather than a difference in shape.

3 Raw Citation Count 1980-2006

Table S5: Student's t-Test comparing means of Randomized to Empirical distributions of concordance values using raw citation counts (1980-2006).

nyears	All	Maximum	Mean	Median	Minimum
1	0.01**	0.02**	0.03***	0.02***	0.00
2	0.01***	0.02***	0.03***	0.02***	0.00
3	0.01***	0.01***	0.02***	0.02***	0.00
4	0.01***	0.02***	0.02***	0.02***	0.00
5	0.01***	0.01***	0.02***	0.02***	0.00
Inf	0.00	0.01*	0.00	-0.02***	-0.03***

Entries are structured as [statistic]$^{\text{[significance]}}$, where the statistic is Student's t and significance is indicated by $*$, $**$, and $***$ to signify $p < 0.05$, $p < 0.01$, and $p < 0.001$, respectively. A positive value indicates that the randomized distribution has a higher mean than the empirical distribution, *i.e.* higher productivity is associated with higher citation rates more often in the randomized records than in the empirically observed ones.

Table S6: Kolmogorov-Smirnov Test comparing Randomized to Empirical distributions of concordance values using raw citation counts (1980-2006).

Transformation	nyears	All	Maximum	Mean	Median	Minimum
none	1	0.05*	0.05*	0.08***	0.05*	0.03
	2	0.11***	0.09***	0.12***	0.08***	0.02
	3	0.10***	0.07***	0.09***	0.07***	0.01
	4	0.12***	0.09***	0.09***	0.08***	0.03
	5	0.08***	0.08***	0.10***	0.07***	0.04
	Inf	0.12***	0.06**	0.06**	0.11***	0.16***
shifted mean	1	0.03	0.05*	0.05*	0.05	0.04
	2	0.03	0.04	0.05	0.03	0.05
	3	0.04	0.03	0.03	0.04	0.03
	4	0.05*	0.03	0.03	0.03	0.04
	5	0.04	0.02	0.04	0.03	0.03
	Inf	0.10***	0.03	0.05	0.05*	0.03
mean 0, sd 1	1	0.03	0.05*	0.05*	0.05	0.04
	2	0.03	0.04	0.05	0.03	0.05
	3	0.03	0.03	0.03	0.04	0.03
	4	0.04	0.03	0.03	0.03	0.03
	5	0.03	0.02	0.03	0.03	0.03
	Inf	0.04	0.03	0.03	0.03	0.02

Entries are structured as [statistic]$^{\text{[significance]}}$, where the statistic is the Kolmogorov-Smirnov test statistic and significance is indicated by $*$, $**$, and $***$ to signify $p < 0.05$, $p < 0.01$, and $p < 0.001$, respectively. The distribution of concordance values being compared is generated from all possible pairings of papers published within "nyears" of each other ("All") or just the "Maximum", "Mean", "Median", or "Minimum" citation rate in each year being compared between years. The distributions being compared are untransformed ("none"), have the randomization means shifted according to the difference identified by a Student's t-Test (see Table S5), or have both distributions rescaled to have means of 0 and standard deviations of 1.

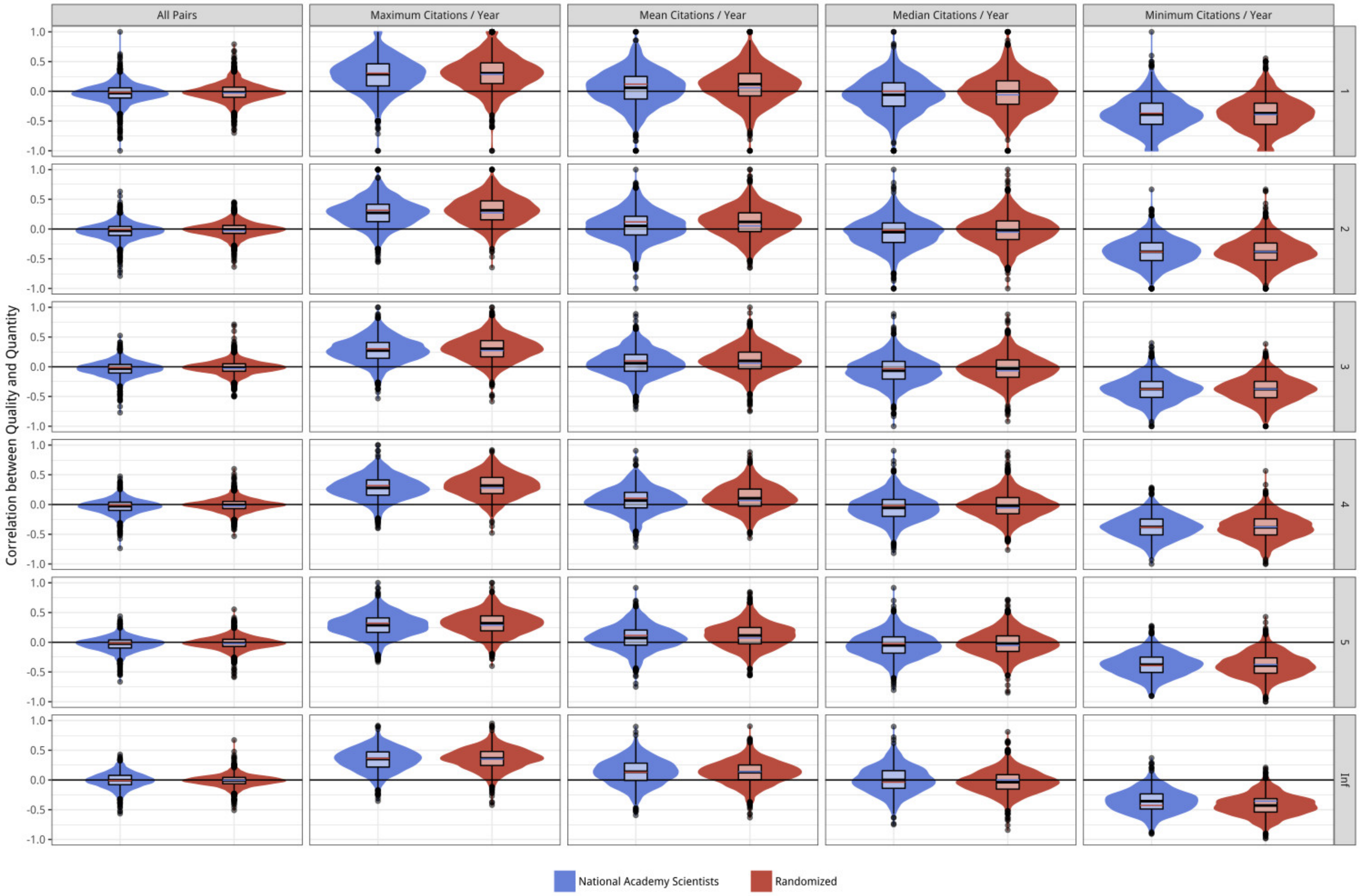

Figure S6: As Fig. S1, but using raw citation counts rather than log citations as the metric of quality. Colored lines in box-plots indicate the location of the median of the complementary box-plot for ease of comparison. Note that a sliding window of infinite width ("Inf") is equivalent to comparing all possible pairs, regardless of temporal distance. The "All Pairs" column refers to running the analysis on all possible pairs of papers with no reduction via summary statistics.

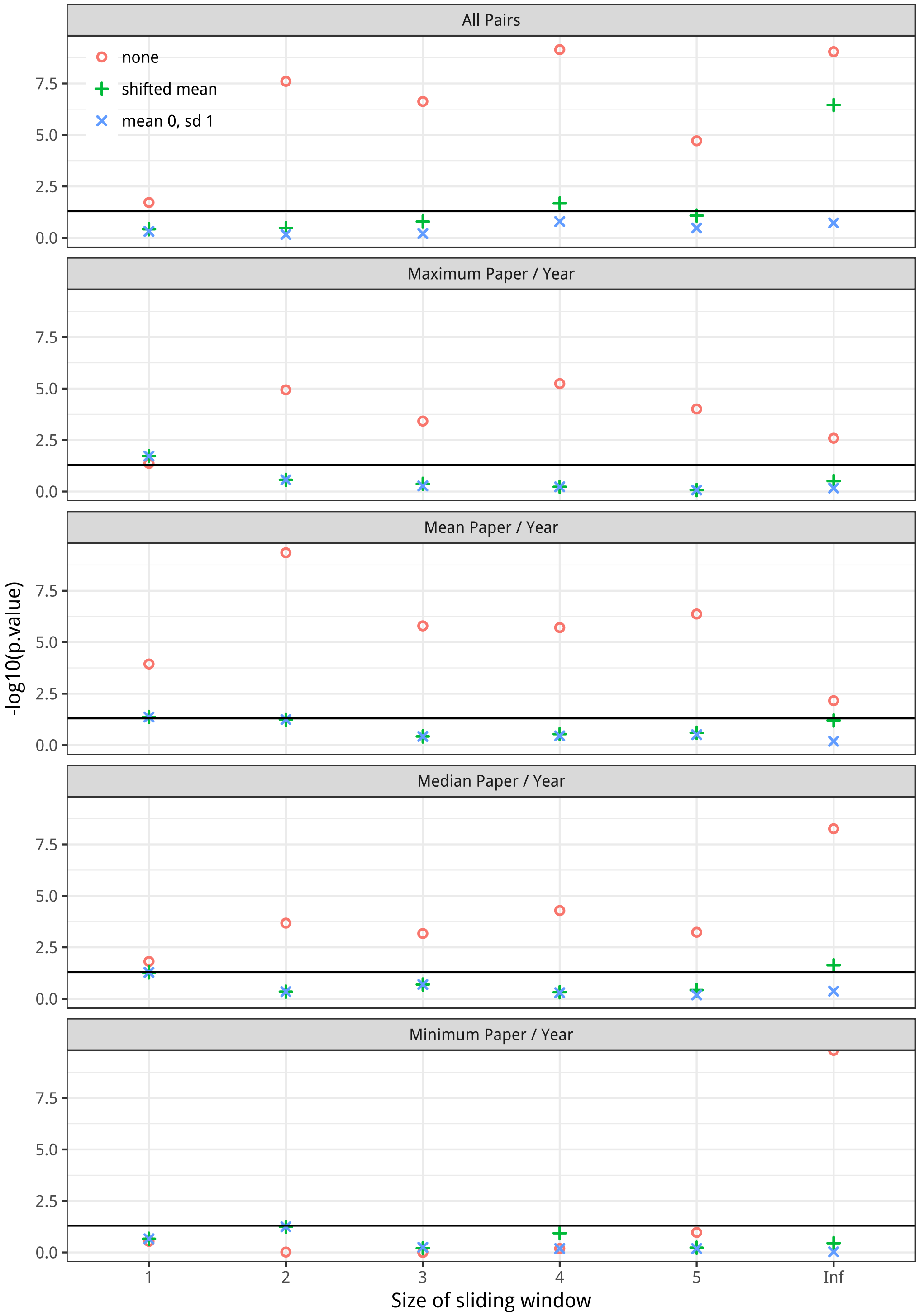

Figure S7: As Fig. S2, but using raw citation counts, rather than log citations as the metric of quality. Trends in the significance level of Kolmogorov-Smirnov Test statistics with increasing sliding window size. Color/shape correspond to the transformation (if any) applied to the data prior to analysis. The facets indicate which pairs are considered (all possible pairs or just one "representative" value per year). Points below the horizontal black line are insignificant (p-value > 0.05). The rapid decline of significance with shifting of the mean and re-scaling suggest the bulk of the difference observed in the distributions is due to a difference in mean, rather than a difference in shape.

4 Log Citation Count 2000-2015

Table S7: Student's t-Test comparing means of Randomized to Empirical distributions of concordance values using log citation counts (2000-2015).

nyears	All	Maximum	Mean	Median	Minimum
1	0.00	0.00	0.01*	0.01	0.00
2	0.01**	0.02**	0.01**	0.01	-0.01
3	0.01***	0.01**	0.01	0.00	-0.02***
4	0.01*	0.02***	0.01	0.01*	-0.02***
5	0.00	0.02***	0.01	0.01	-0.03***
Inf	-0.01	0.04***	-0.01**	-0.01*	-0.06***

Entries are structured as [statistic][significance], where the statistic is Student's t and significance is indicated by $*$, $**$, and $***$ to signify $p < 0.05$, $p < 0.01$, and $p < 0.001$, respectively. A positive value indicates that the randomized distribution has a higher mean than the empirical distribution, *i.e.* higher productivity is associated with higher citation rates more often in the randomized records than in the empirically observed ones.

Table S8: Kolmogorov-Smirnov Test comparing Randomized to Empirical distributions of concordance values using log citation counts (2000-2015).

Transformation	nyears	All	Maximum	Mean	Median	Minimum
none	1	0.07**	0.02	0.06*	0.04	0.03
	2	0.09***	0.05*	0.06*	0.05	0.04
	3	0.09***	0.05*	0.04	0.04	0.07**
	4	0.07**	0.07**	0.05	0.04	0.09***
	5	0.07**	0.09***	0.04	0.04	0.11***
	Inf	0.20***	0.19***	0.10***	0.11***	0.21***
shifted mean	1	0.04	0.08***	0.07**	0.10***	0.10***
	2	0.04	0.03	0.05*	0.05	0.03
	3	0.04	0.03	0.03	0.06*	0.05
	4	0.06**	0.04	0.03	0.04	0.04
	5	0.08***	0.03	0.03	0.04	0.02
	Inf	0.18***	0.08***	0.08***	0.09***	0.05
mean 0, sd 1	1	0.04	0.08***	0.06*	0.10***	0.10***
	2	0.03	0.03	0.05*	0.05	0.03
	3	0.02	0.03	0.03	0.06*	0.05
	4	0.05	0.03	0.03	0.04	0.04
	5	0.05	0.02	0.02	0.04	0.02
	Inf	0.06*	0.03	0.02	0.03	0.03

Entries are structured as [statistic][significance], where the statistic is the Kolmogorov-Smirnov test statistic and significance is indicated by $*$, $**$, and $***$ to signify $p < 0.05$, $p < 0.01$, and $p < 0.001$, respectively. The distribution of concordance values being compared is generated from all possible pairings of papers published within "nyears" of each other ("All") or just the "Maximum", "Mean", "Median", or "Minimum" citation rate in each year being compared between years. The distributions being compared are untransformed ("none"), have the randomization means shifted according to the difference identified by a Student's t-Test (see Table S7), or have both distributions rescaled to have means of 0 and standard deviations of 1.

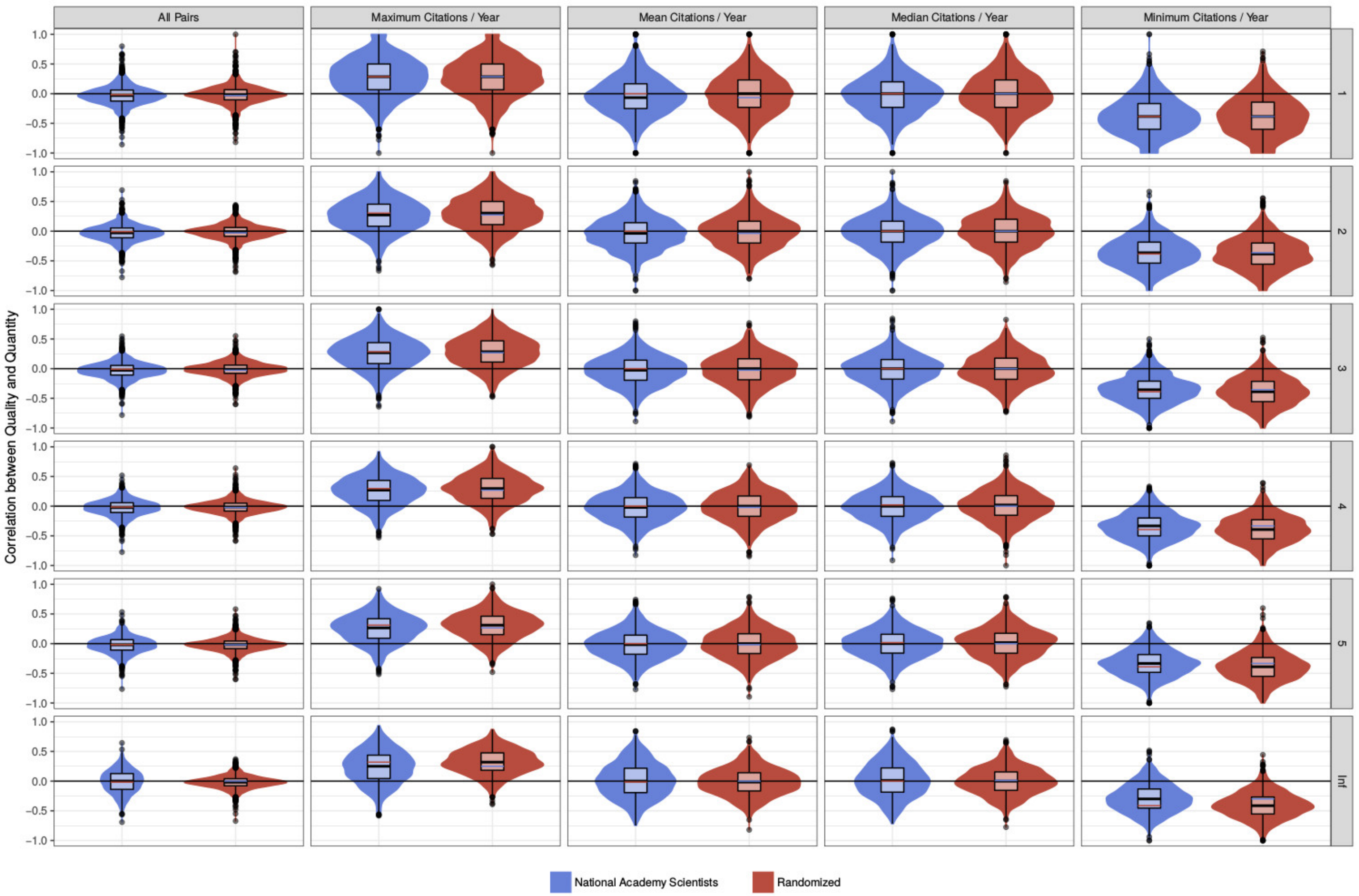

Figure S8: As Figs. S1 and S8, and Figure 1 in the main text, but for all sliding window sizes and for all possible pairings of papers as well as pairings of summary statistics and 2000-2015. Colored lines in box-plots indicate the location of the median of the complementary box-plot for ease of comparison. Note that a sliding window of infinite width ("Inf") is equivalent to comparing all possible pairs, regardless of temporal distance. The "All Pairs" column refers to running the analysis on all possible pairs of papers with no reduction via summary statistics.

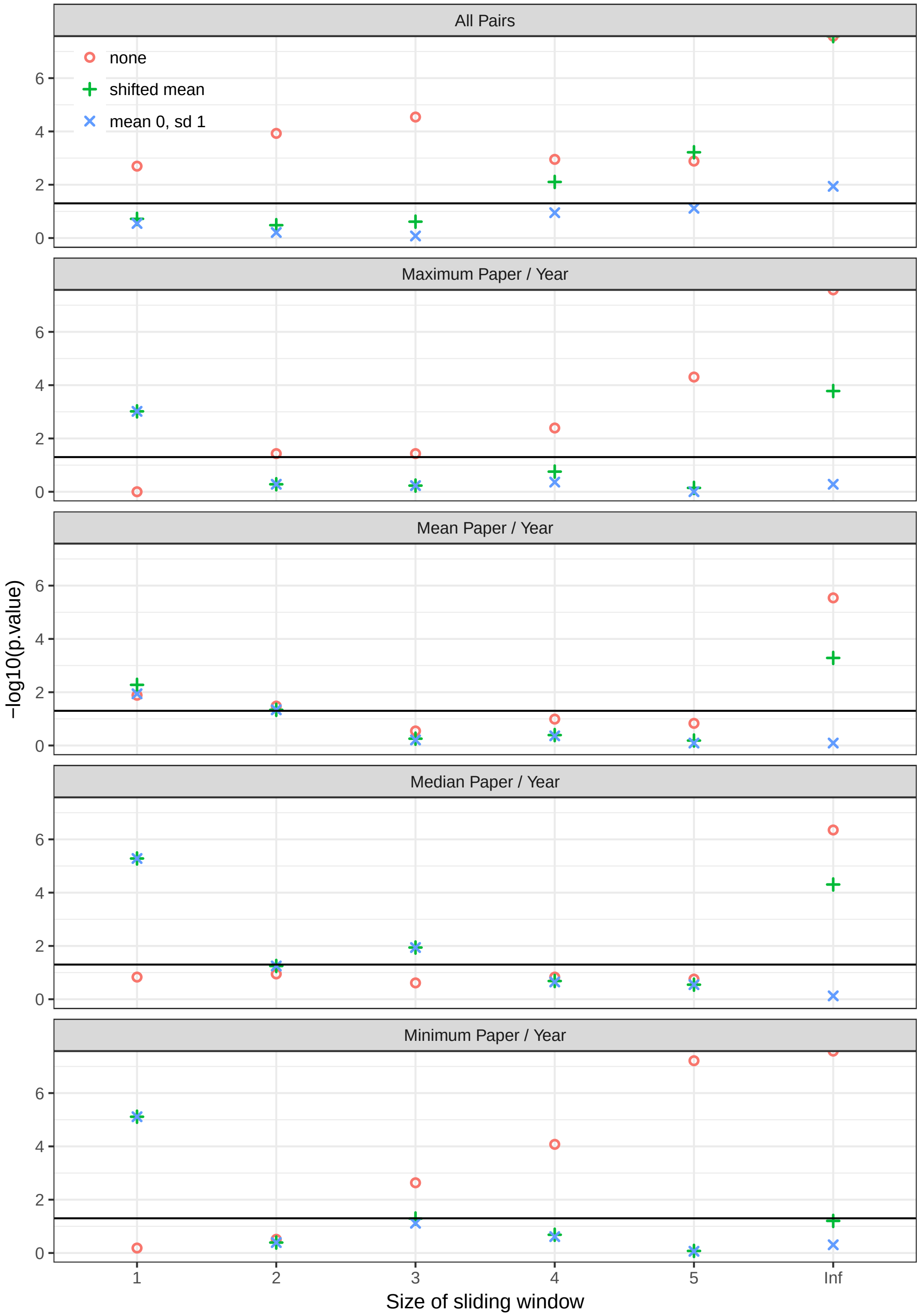

Figure S9: As Fig. S2, but over the timespan of 2000-2015. Trends in the significance level of Kolmogorov-Smirnov Test statistics with increasing sliding window size. Color/shape correspond to the transformation (if any) applied to the data prior to analysis. The facets indicate which pairs are considered (all possible pairs or just one "representative" value per year). Points below the horizontal black line are insignificant (p-value > 0.05). The rapid decline of significance with shifting of the mean and re-scaling suggest the bulk of the difference observed in the distributions is due to a difference in mean, rather than a difference in shape.

5 Productivity Through Time 1948-2015

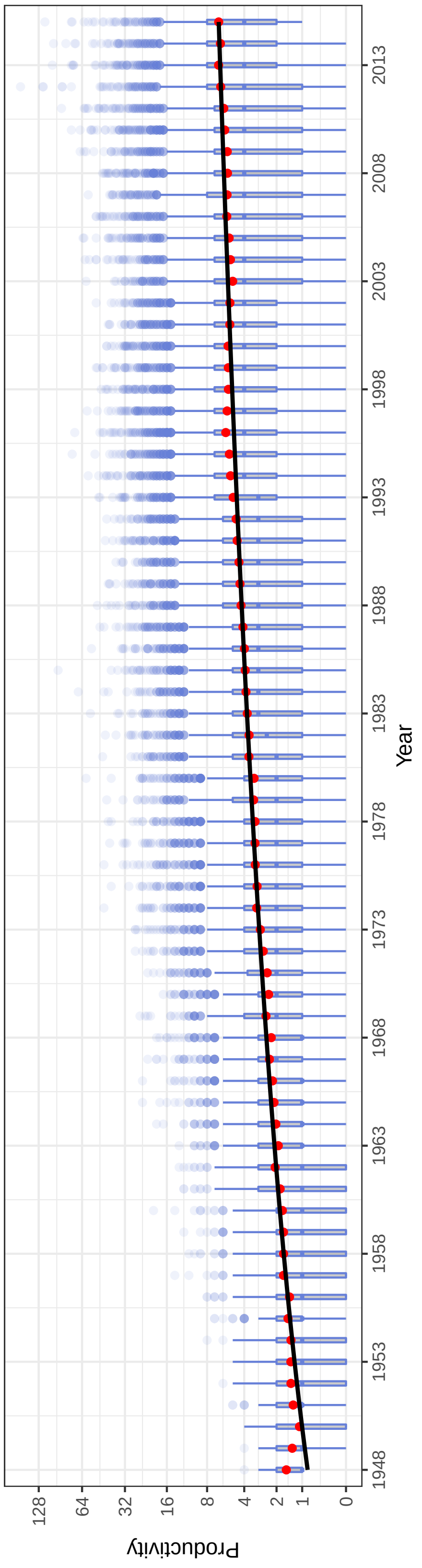

Figure S10: Annual productivity of each author in the full dataset (excluding years earlier than 1948 due to few authors). Each box-plot indicates the distribution of productivities for each year and the red points indicate the mean productivity each year. The black line is the best fit linear model to the data, with a slope of 0.084, $p \ll 0$ and $R^2 = 0.053$. The curve in the linear model fit results from the log-scaling the vertical axis.
